# The Hidden Burden: Systemic and Individual Factors Driving Extensive Care in Personality Disorders

**DOI:** 10.1192/j.eurpsy.2025.549

**Published:** 2025-08-26

**Authors:** P. Sand, F. G. Di Leone

**Affiliations:** 1 Department of psychology, University of Gothenburg; 2 Affective psychiatry, Sahlgrenska Univeristy Hospital; 3 Institute of Neuroscience and Physiology, University of Gothenburg, Gothenburg, Sweden

## Abstract

**Introduction:**

Individuals with personality disorders (PD) are overrepresented in emergency psychiatric settings, leading to both frequent and prolonged hospitalizations, compulsory care and high healthcare costs.

**Objectives:**

1. To delineate the extensive care needs of participants, (2) To identify commonalities in their life situations, comorbidities, and treatment interventions, and (3) To investigate patient experiences with the healthcare system to uncover precipitating and contributing factors to extensive care needs.

**Methods:**

The quantitative study utilized a descriptive cross-sectional design, drawing on data from 61 individuals diagnosed with PD who exhibited extensive care needs, operationally defined as having made five or more visits to the emergency department, at least three hospitalizations, or a total of 60 hospitalization days during 2023. The qualitative study involved in-depth interviews with 11 of these individuals, analyzed using interpretative phenomenological analysis (IPA) to explore patient experiences and needs.

**Results:**

The findings indicate that individuals with comorbid conditions experienced significantly more frequent and prolonged hospitalizations. However, there was no significant difference in the number of emergency department visits between the two groups. Qualitative insights revealed four main themes with two focusing on precipitating factors, and two addressing perpetuating factors. The results indicated that emergency department visits were frequently driven by emotional distress and social isolation. Patients described their treatment experiences as impersonal and lacking direction, with frequent medication adjustments resulting in adverse effects and heightened feelings of hopelessness.

**Conclusions:**

The study highlights that extensive care needs among individuals with personality disorders (PD) arise from a combination of high psychopathological burden and social marginalization. Many high-care consumers rely on hospitalization as their primary support, reflecting a psychiatric system ill-equipped to meet their complex needs. This mismatch leads to patient dissatisfaction and a revolving-door effect, where repeated hospitalizations further detach patients from their social environments, increasing the risk of continued high care consumption. Overmedicalization and frequent hospitalizations exacerbate social isolation, fueling a cycle of increased care dependency. To address these challenges, the study proposes targeted interventions, including: (1) early assessment of risk factors such as social isolation, comorbid conditions, and lack of social roles, (2) promoting alternative forms of hospitalization, (3) implementing a stepped care model for crisis management, and (4) improving comorbidity management and medication oversight.

**Disclosure of Interest:**

None Declared

